# Laparoscopic transhiatal surgery for an epiphrenic esophageal diverticulum derived from a jackhammer esophagus: a case report

**DOI:** 10.1186/s40792-020-00900-2

**Published:** 2020-06-29

**Authors:** Atsuro Fujinaga, Tomotaka Shibata, Tsuyoshi Etoh, Kazuhiro Tada, Kosuke Suzuki, Kohei Nishiki, Katsuhiro Ogawa, Yohei Kono, Takahiro Hiratsuka, Tomonori Akagi, Yoshitake Ueda, Manabu Toujigamori, Hidefumi Shiroshita, Norio Shiraishi, Masafumi Inomata

**Affiliations:** 1 Department of Gastroenterological and Pediatric Surgery, Oita University Faculty of Medicine, Idaigaoka 1-1, Hasama-machi, Oita, 879-5593 Japan; 2 Department of Gastroenterological Surgery, Oita Nakamura Hospital, Oita, Japan; 3 Department of Comprehensive Surgery for Community Medicine, Oita University Faculty of Medicine, Oita, Japan

**Keywords:** Jackhammer esophagus, Epiphrenic esophageal diverticulum, Myotomy

## Abstract

**Background:**

An esophageal diverticulum is rare and is frequently associated with esophageal motility disorders. Jackhammer esophagus is also rare, is characterized by esophageal hypercontraction, and comprises 4.1% of esophageal motility disorders. Here, we report a case of a patient successfully treated by laparoscopic transhiatal surgery for an epiphrenic esophageal diverticulum derived from a jackhammer esophagus diagnosed with high-resolution manometry (HRM).

**Case presentation:**

The patient was a 78-year-old man who presented to the hospital with dysphagia. A diverticulum was detected in the lower part of his esophagus by upper gastrointestinal endoscopy. HRM was performed to investigate esophageal motility disorders. His integrated relaxation pressure was normal at 25.9 (< 26) mmHg, but his distal contractile integral (DCI) was very high at 21,464 (1500–13,000) mmHg s cm. Esophageal peristalsis was preserved. Therefore, the patient was diagnosed as having an epiphrenic esophageal diverticulum derived from a jackhammer esophagus for which laparoscopic transhiatal diverticulectomy and Heller-Dor procedure were performed. The postoperative course was uneventful. His symptoms improved, and the level of DCI also returned to a normal level of 3867 mmHg s cm at 2 months after the operation.

**Conclusion:**

Laparoscopic transhiatal diverticulectomy and esophagomyotomy can be useful procedures for an epiphrenic esophageal diverticulum derived from a jackhammer esophagus due to their lower invasiveness.

## Background

Esophageal diverticulum has been reported to occur at a frequency of 1% among all gastrointestinal diverticula [1]. The epiphrenic esophageal diverticulum, a pulsion diverticulum caused by the pressure of esophageal muscle movement applied to the weak part of the lower esophagus, is often associated with esophageal motility disorders [2, 3]. In recent years, high-resolution manometry (HRM) has been developed as a manometric method to assess the esophagus. In the new Chicago Classification, the diagnostic criteria for esophageal motility disorder were revised based on HRM techniques.

Jackhammer esophagus is characterized by esophageal hypercontraction and is a rare disorder among the esophageal motility disorders [4–6]. In the 2012 Chicago Classification, jackhammer esophagus was identified as a subtype of nutcracker esophagus (NE) [7]. We present a patient who successfully underwent laparoscopic transhiatal surgery for an epiphrenic esophageal diverticulum derived from a jackhammer esophagus diagnosed with HRM.

## Case presentation

**Fig. 1:**
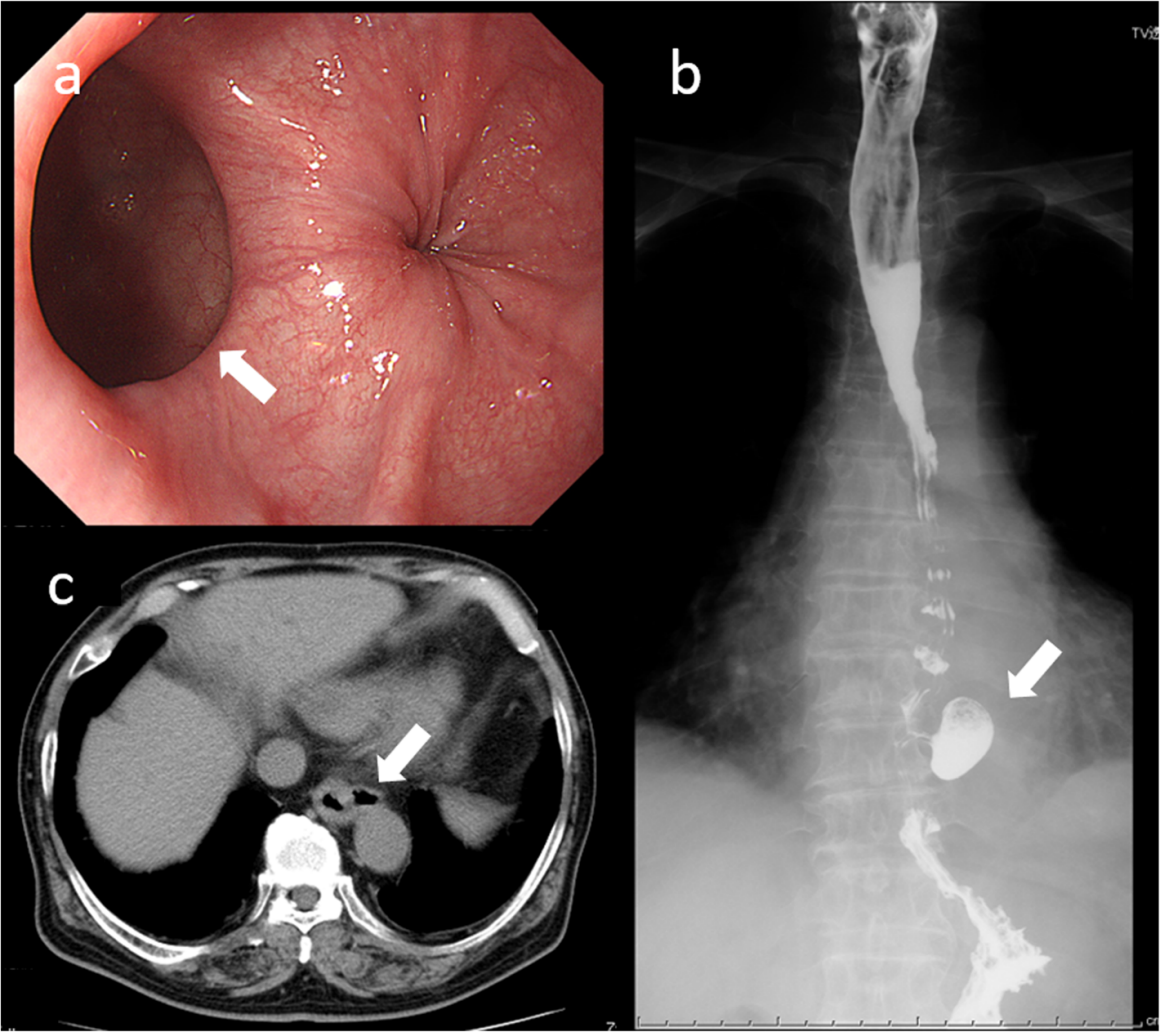
**a** Upper gastrointestinal endoscopy showed a 31-mm diverticulum on the left wall of the lower esophagus (arrow). **b** A barium esophagogram showed a diverticulum in the lower esophagus 50 mm above the EGJ (arrow). **c** Chest CT revealed a diverticulum on the left wall in the lower esophagus with no esophageal masses (arrow)

A 78-year-old Japanese man presented to the hospital with dysphagia. His symptom remained even after treatment with proton pump inhibitors. Upper gastrointestinal endoscopy found a 31-mm diverticulum on the left wall in the lower part of the esophagus (Fig. 1a). The mucosa of the diverticulum was normal. Moreover, a barium esophagogram showed a diverticulum in the lower esophagus 50 mm above the esophagogastric junction (EGJ) and no obstruction of contrast at the lower esophageal sphincter (Fig. 1b). Chest computed tomography (CT) also revealed a diverticulum on the left wall in the lower esophagus (Fig. 1c). There was no history of esophageal motility disorder in the patient’s family.

**Fig. 2:**
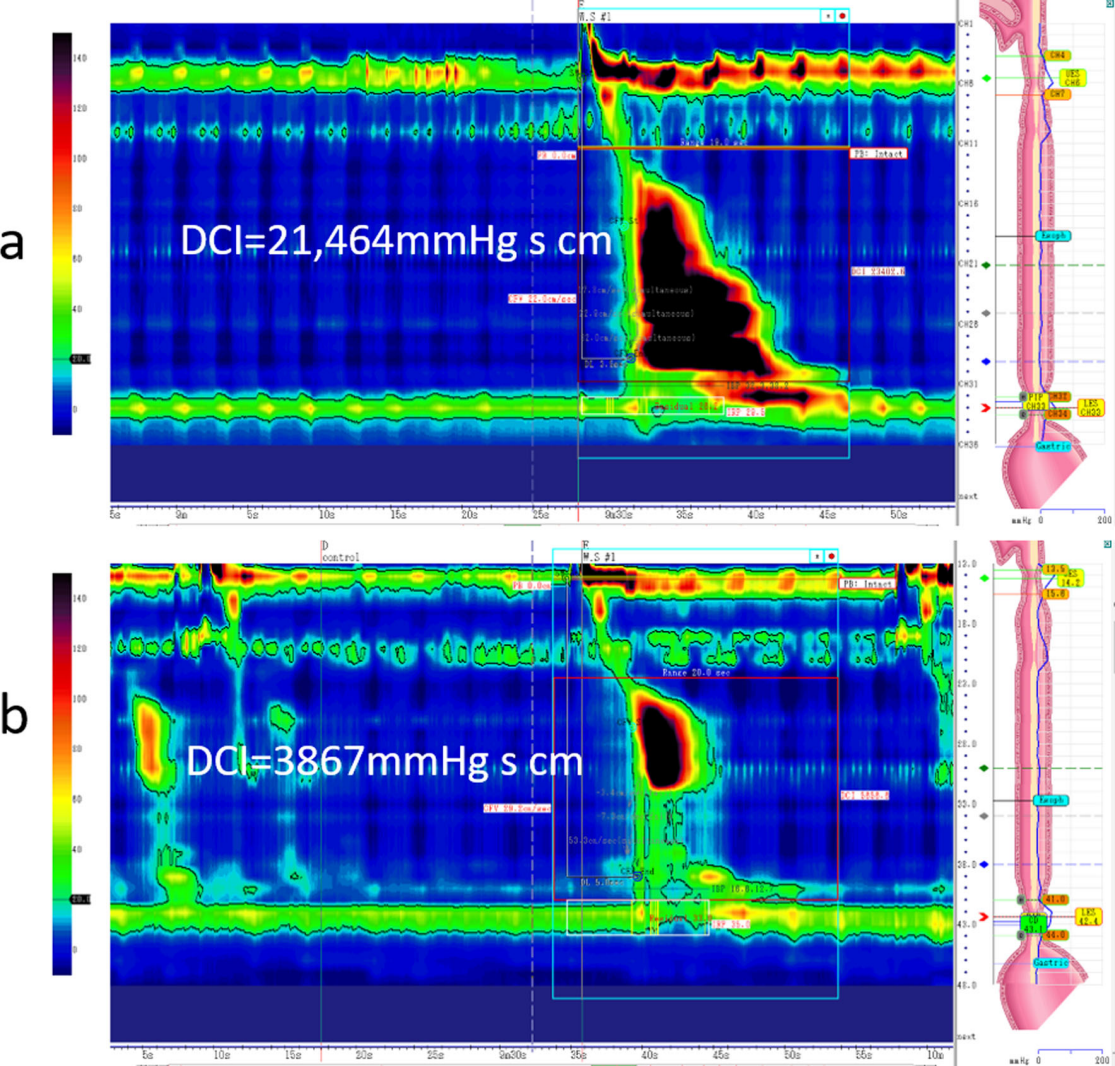
**a** Preoperative HRM. DCI was high at 21,464 mmHg s cm. **b** Postoperative HRM. The level of DCI improved to normal at 3867 mmHg s cm

Upon admission, his height, weight, and body mass index were 158 cm, 66.2 kg, and 26.5 kg/m^2^, respectively. Laboratory tests showed no anemia and revealed normal liver and kidney functions. HRM by Starlet (STAR MEDICAL, Tokyo, Japan) was performed to determine the presence of any esophageal motility disorder. The HRM catheter was inserted transnasally and placed in the optimal position in the esophagus, and the distal sensor was placed 2 to 3 cm below the diaphragm. A standard HRM protocol consists of a baseline quiescent period lasting at least 30 s, followed by a series of 10 5-mL swallows of room temperature water with the patient in the supine or reclined position [8]. The patient’s integrated relaxation pressure (IRP) was normal at 25.9 (normal < 26) mmHg, whereas his distal contractile integral (DCI) was very high at 21,464 (normal 1500–13,000) mmHg s cm. Esophageal peristalsis was preserved (Fig. 2a). Therefore, the patient was diagnosed as having an epiphrenic esophageal diverticulum derived from a jackhammer esophagus. Then, a laparoscopic diverticulectomy and esophagomyotomy (Heller procedure) were performed.

**Fig. 3:**
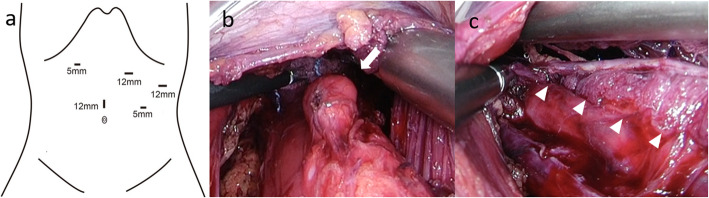
**a** Trocar arrangement in this procedure. **b** An esophageal diverticulum was observed on the left side of the esophageal wall 5 cm above the EGJ (arrow). **c** Esophagomyotomy of the front wall was performed from the EGJ to 10 cm above the EGJ

After taping the abdominal esophagus, we dissected tissues along the esophagus to the oral side. An esophageal diverticulum was observed on the left side of the esophageal wall 5 cm above the EGJ (Fig. 3b). The diverticulectomy was performed using a linear stapler while confirming the lumen of the esophagus under observation with intraoperative esophagoscopy, and the staple line was reinforced. An esophagomyotomy of the front wall was also performed from the EGJ to 10 cm above the EGJ (Fig. 3c). As the patient’s IRP level was normal from the findings of HRM, we did not perform gastric myotomy. Finally, the Dor procedure was performed. The operation time and blood loss were 270 min and 5 mL, respectively. The diverticulum specimen was 30 mm × 20 mm in diameter with normal mucosa. The diverticulum wall lacked a proper muscular layer of the esophagus. The pathological diagnosis was a pseudodiverticulum with no evidence of malignancy.

The patient’s postoperative course was uneventful, and he was discharged from the hospital 12 days after surgery. At 2 months after surgery, his symptoms had improved, and his DCI had returned to a normal level of 3867 mmHg s cm (Fig. 2b).

## Discussion

Esophageal diverticulum derived from a jackhammer esophagus is considered to be a very rare disease that is difficult to diagnose. In the present case, from the findings of HRM, we could diagnose the patient as having a jackhammer esophagus. After the laparoscopic transhiatal diverticulectomy and esophagomyotomy, his symptoms and DCI level improved.

In recent years, HRM has been developed as a method of esophageal manometry. HRM uses a high-resolution catheter to transmit intraluminal pressure data that is subsequently converted into dynamic esophageal pressure topography plots [9]. Metric data from these plots are synthesized to yield an esophageal motility diagnosis according to the Chicago Classification version 3.0, a formal analytic scheme for esophageal motility disorders [7]. In the 2012 Chicago Classification, jackhammer esophagus was identified as a subtype of NE and was defined as an esophageal motility disorder characterized by a DCI greater than 8000 mmHg s cm with normal IRP in at least two of ten swallows on manometry. Jackhammer esophagus is rare, occurring in only 4.1% of patients undergoing HRM in a series of 2000 patients [6]. We could make an accurate diagnosis from the findings of HRM, and we thought HRM was useful to diagnose esophageal motility disorders.

Esophageal diverticulum has been reported to occur at a frequency of 1%, the lowest among all gastrointestinal diverticula [1]. The types of esophageal diverticulum include pharyngoesophageal diverticulum (Zenker’s diverticulum), middle esophageal diverticulum, and epiphrenic esophageal diverticulum, which occurs at a frequency of 11% among esophageal diverticula [1]. Epiphrenic esophageal diverticulum is a pulsion diverticulum caused by the pressure of esophageal muscle movement applied to the weak part of the lower esophagus [2]. Surgical treatment should be actively performed for those with severe symptoms such as dysphagia and pain and those with growing or poor drainage of content in the diverticulum [3]. The esophageal diverticulum is often associated with esophageal motility disorders [3]. Khullar et al. have reported favorable results of diverticulectomy and esophagomyotomy for esophageal diverticula derived from jackhammer esophagus [10]. They suggested that surgery is necessary not only for diverticula but also for esophageal motility disorder.

Endoscopic or surgical esophagomyotomy can be performed for esophageal motility disorder. There are only few reports of per-oral endoscopic myotomy (POEM) and surgical myotomy for non-achalasia motility disorders. Hoppo et al. reported that POEM was performed in 5 cases of jackhammer esophagus, 1 case of diffuse esophageal spasm (DES), and 2 cases of NE, with improvement of 75% for dysphagia and 80% for chest pain [11]. Schlottmann et al. performed surgical myotomy in 19 cases of DES and 12 cases of NE, of which 15 of the DES (80%) and 6 of the NE (50%) cases improved [12]. To the best of our knowledge, there are only two reports of surgical esophagomyotomy performed for jackhammer esophagus, and the results were good [10, 13]. Therefore, esophagomyotomy could be effective for jackhammer esophagus. Rossetti et al. reported that esophagomyotomy can be performed just above the lower esophageal sphincter (LES) in patients with epiphrenic esophageal diverticulum with normal function of the LES [14, 15]. As the patient’s IRP level was normal from the findings of HRM, we did not perform gastric myotomy in this patient.

It is reported that laparoscopic transhiatal manipulation is possible up to the middle esophagus [16]. With the progress in laparoscopic surgery [17], laparoscopic transhiatal esophagomyotomy for esophageal motility disorder even in the superior portion could be performed safely. Conventionally, following esophageal surgery, there are possibilities of postoperative respiratory complications due to the adverse effects of thoracotomy and associated one-lung ventilation [18]. However, a transhiatal procedure without thoracotomy is less invasive and has fewer postoperative complications [19]. There is no consensus of the length of esophagomyotomy in patients with esophageal diverticulum [20]. Therefore, both the findings of HRM and possible manipulations should be considered to determine the length of esophagomyotomy. The extent of the lesions, including the diverticulum, was mainly in the middle and lower esophagus; therefore, we believed that laparoscopic transhiatal diverticulectomy and esophagomyotomy from the EGJ to 10 cm above the EGJ would improve esophageal diverticulum and motility disorder. Since the location of the diverticulum was typical of the left esophageal wall, esophagomyotomy of the front wall was performed safely. After surgery, the patient’s DCI level improved from 21,464 to 3867 mmHg s cm along with his symptoms. The findings in our patient showed that laparoscopic transhiatal diverticulectomy and esophagomyotomy can be useful procedures.

## Conclusions

Esophageal motility disorder, which should be treated from the lower to the superior portion of the esophagus, could be treated by the laparoscopic transhiatal approach. The laparoscopic procedures of transhiatal diverticulectomy and esophagomyotomy are less invasive and can be useful for treating an epiphrenic esophageal diverticulum derived from a jackhammer esophagus.

## Data Availability

The data are not available for public access because of patient privacy concerns but are available from the corresponding author on reasonable request.

## Abbreviations

HRM: High-resolution manometry
NE: Nutcracker esophagus
EGJ: Esophagogastric junction
CT: Computed tomography
IRP: Integrated relaxation pressure
DCI: Distal contractile integral
POEM: Per-oral endoscopic myotomy
DES: Diffuse esophageal spasm
LES: Lower esophageal sphincter
